# Supplementary material for: Antimicrobial and Anti-Biofilm Activity of Polymyxin E Alone and in Combination with Probiotic Strains of Bacillus subtilis KATMIRA1933 and Bacillus amyloliquefaciens B-1895 against Clinical Isolates of Selected Acinetobacter spp.: A Preliminary Study
Source: Pathogens. 2021 Dec 2;10(12):1574. doi: 10.3390/pathogens10121574 (PMC8707300; doi:10.3390/pathogens10121574)
Supplement: Supplementary file 1 [file pathogens-10-01574-s001.zip › pathogens-1472672-supplementary.pdf]

Table S1. The diameters of inhibition zones; interpretive standards for the selected *Acinetobacter* spp. strains according to (CLSI, 2020).

Antibiotic Discs	Code	Diameter of Inhibition Zone (mm)		
		Susceptible	Intermediate	Resistant
Amikacin	AK	≥17	15–16	14≤
Cefotaxime	CTX	≥23	15–22	14≤
Meropenem	MEM	≥18	15–17	14≤
Trimethoprim-Sulfamethoxazole	SXT	16≥	11–15	10≤
Polymyxin E	PME	-	-	-
Cefoxitin	Fox	≥21	14–20	≤13
